# Association of breast arterial calcification and osteoporosis in Turkish women

**DOI:** 10.12669/pjms.312.6120

**Published:** 2015

**Authors:** Nesrin Atci, Eda Elverici, Raziye Keskin Kurt, Derya Ozen, Ebru Unlu, Levent Araz

**Affiliations:** 1 Nesrin Atci, MD, Department of Radiology, Mustafa Kemal University Medical School, Hatay, Turkey; 2 Eda Elverici, MD, Numune Education and Research Hospital, Department of Radiology, Ankara, Turkey; 3 Raziye Keskin Kurt, MD, Department of Obstetrics and Gynecology, Mustafa Kemal University Medical School, Hatay, Turkey; 4 Derya Ozen, MD, Numune Education and Research Hospital, Department of Radiology, Ankara, Turkey; 5 Ebru Unlu, MD, Afyon Kocatepe University Medical School, Department of Radiology, Afyon, Turkey; 6 Levent Araz, MD, Numune Education and Research Hospital, Department of Radiology, Ankara, Turkey

**Keywords:** Breast arterial calcification, Bone mineral density, Osteopenia, Osteoporosis, Menopause

## Abstract

**Objective:**

Breast arterial calcification (BAC), medial calcific sclerosis of small to medium-sized muscular arteries, is a benign finding of mammographic evaluation. Previous studies have shown the relationships between BAC and systemic disorders such as cardiovascular disease, diabetes mellitus and hypertension. The aim of this study was to determine the association between reduced bone mineral density and BAC.

**Methods:**

The study population consisted of 567 women who had both mammography and bone mineral density evaluation. BAC (+) and BAC (-) women were compared for age, body mass index, postmenopausal duration, number of deliveries, breastfeeding duration, DM, HT, lipid treatment, osteopenia, and osteoporosis.

**Results:**

BAC was seen in mammographic evaluation of 179 women and 388 subjects without BAC accepted as the control group. There was a statistically significant relationship between age, postmenopausal duration, number of deliveries, history of DM, HT, lipid treatment and BAC. While the prevalence of osteopenia was higher in control group (52.8%), the rate of osteoporosis (48.7%) was higher in group with BAC.

**Conclusion:**

There was statistically significant relationship between BAC and osteoporosis in postmenopausal women. Determination of BAC in routine screening mammography might be helpful in both identifying women with risk of cardiovascular disease and osteoporosis.

## INTRODUCTION

Breast arterial calcification (BAC) is a benign finding of mammographic evaluation with a high prevalence on routine screening.^1^ BAC is recognized as medial calcific sclerosis of small to medium-sized muscular arteries of breast, and occurs more frequently in older women without association with breast cancer.^2^ Previous studies have shown the relationships between BAC and cardiovascular diseases (CVD), diabetes mellitus (DM), and hypertension (HT).^3^ It has speculated that BAC results from a complex process of biomineralization resembling bone formation.^4^ Nevertheless, in English literature there is only one study reporting the relationship between bone mineral density (BMD) and BAC. In their study, Reddy et al.^4^ claimed that women with BAC were more likely to have reduced BMD as compared with women without BAC. In the present study, we aimed to evaluate the association with BAC and osteoporosis.

## METHODS

### Study Population

This study involved all the women who concurrently visited the Ankara Numune Training and Research Hospital Department of Radiology for routine postmenopausal control between March and July 2009. Study protocol respected to the Helsinki Declaration principles, and our institutional ethics review board approved it. In this retrospective study, 567 subjects were enrolled. Age, height, weight, body mass index, menopause duration, number of deliveries, breastfeeding duration, presence of diabetes mellitus (DM) or hypertension (HT), lipid treatment were recorded for each subject. Body mass index was calculated by the ratio of weight (kg) and height square (m^2^).

### Mammography

The mammography images of the subjects were obtained with full area digital mammography device (Selenia digital mammography system-Lorad HOLOGIC). In all the subjects, 4 images were obtained for both breasts in standard craniocaudal (CC) and mediolateral oblique (MLO) positions. BAC was defined based on the classification by Sickles (5), and breast pattern classification was made according to BI-RADS (Breast Imaging Reporting And Data System) defined by American College of Radiology in BI-RADS atlas of 2003. The evaluation was performed by the same radiologists (N.A) blinded to the clinical and bone density evaluation. Intraobserver variability of BAC assessment was performed by evaluating 40 mammographies (20 with BAC, and 20 without BAC) 5 days apart.

### BMD Evaluation

Dual-energy X-ray absorptiometry method (HOLOGIC Discovery W, USA) was used for evaluation of osteoporosis. The measurements were conducted from the anterior-posterior lumbal vertebra (L1-4) and right hip area of the subjects. BMD definitions were based on T scoring according to WHO classification.^5^ T score of – 1.0 and above was considered as normal; -1.0 to -2.5 as osteopenia, and under -2.5 as osteoporosis (national osteoporosis foundation guided). The classification of bone mineral density was made on the lowest T-score of the lumbar spine, total hip, or both.

### Statistical Analysis

The data were analyzed with SPSS for Windows 11.5 statistical package program. Continuous variables were expressed as mean ± SD, and categorical variables were expressed as percentages. Analysis of normality of the continuous variables was performed with kolmogorov-Smirnov test. Comparison of categorical and continuous variables between the two groups was performed using the χ^2^ test or Fischer’s exact test, and independent sample t-test, respectively. P<0.05 was considered statistically significant.

## RESULTS

**Fig.1:**
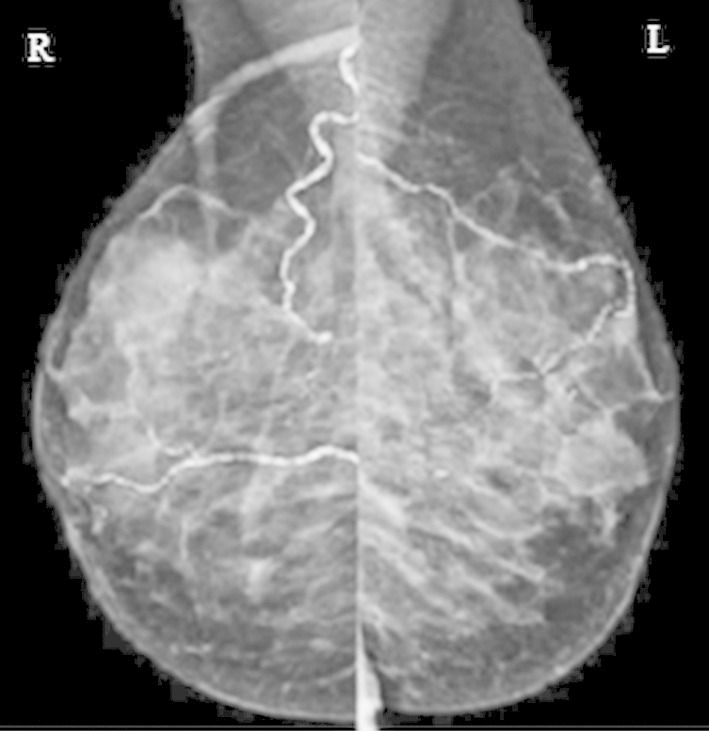
Vascular calcification is widely followed in both breasts mediolateral oblique graphy.

BAC was seen in one or both breasts in mammographic screening [BAC (+)] (Fig.1) of 179 women and 388 subjects without BAC accepted as the control group [BAC (-)]. The clinical, reproductive characteristics of BAC+ and BAC-groups are presented in Table-I. There was a statistically significant difference in the history of DM (21.8% of BAC+ group, 14.4% of BAC-group) (p=0.029), HT (57.3% of BAC+ group, 34.1% of BAC-group) (p<0.001), lipid treatment (20.8% of BAC+ group, 11.4% of BAC-group) (p=0.003) between two groups.

**Table-I:** The Demographic Data of BAC (+) and BAC (-) Groups.

Variables	BAC (+) (n=179)	BAC (-) (n=388)	p
Age (years)	65.3 ± 4.1	55.4 ± 5.1	<0.001
Body Mass Index (kg/m^2^)	36.2 ± 3.1	33.8 ± 2.6	0.817
Menopause Status(years)	12.8 ± 3.4	5.4 ± 2.1	<0.001
Number of deliveries	6.1 ± 2.1	3.6 ± 3.4	<0.001
Breast feeding Time (months)	21±3.1	22±4.1	0.258
Diabetes History	39 (21.8%)	56 (14.4%)	0.029
Lipid Treatment History	37 (20.8%)	44 (11.4%)	0.003
Hypertension History	102 (57.3%)	132 (34.1%)	<0.001
Osteopenia	24 (30.8%)	103 (52.8%)	<0.001
Osteoporosis	38 (48.7%)	35 (17.9%)	<0.001

BAC incidence was statistically significantly higher in those over 60 years of age (p<0.001), and with postmenopausal duration more than 10 years (p< 0.001). The mean number deliveries was statistically significantly higher in BAC+ group than control group (6.1±2.1, and 3.6±3.4 respectively) (p<0.001). No statistically significant difference was found between the two groups for breastfeeding times (p=0.258), and BMI (p=0.817).

**Fig.2:**
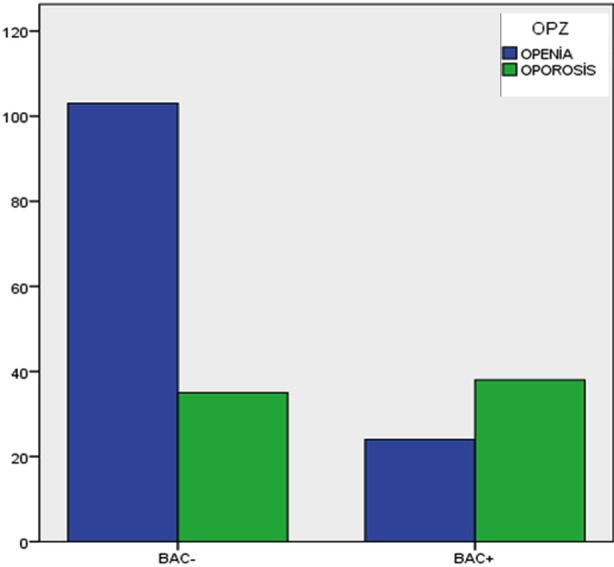
The rates of osteoporosis, and osteopenia within BAC (+) and BAC (-).

The prevalence of osteopenia was higher in BAC (-) group (52.8%), while the rate of osteoporosis (48.7%) was higher in BAC (+) group. The differences between the groups for these parameters were statistically significant (p<0.001). The rates of osteoporosis, and osteopenia within BAC (+) and BAC (-) groups have been presented in Fig.2.

## DISCUSSION

The data of this study reveal a significant relationship between BAC and osteoporosis in elderly women. It was also shown that there was a statistically significant relationship between age, postmenopausal duration, number of deliveries, history of DM, HT, lipid treatment and BAC which was compatible with the previous studies.^1^,^6^

In elderly women, cardiovascular disease and osteoporosis are important and frequent reasons for morbidity and mortality.^1^ Reduced BMD in postmenopausal women constitutes an increased risk factor for acute stroke and cardiovascular disease associated mortality in advanced ages.^4^ Jorgensen et al.^7^ have found that women with low BMD have 4.8 times higher risk of stroke than those with high BMD levels. Similarly, Kiel et al.^8^ have shown a higher incidence of coronary disease in 30-year follow-up of women with low BMD. Markovitz et al.^9^ have reported an independent relationship between BMD and angiography-proven coronary artery stenosis and multiple artery coronary disease. Reduced BMD was also found to be associated with subclinical vascular disease markers (aortic artery calcification, coronary artery calcium score, and carotid artery atherosclerosis…etc). Shulz et al.^10^ have determined a marked age-dependent relationship between bone density and aortic calcification on CT of a group of healthy postmenopausal women. Uyama et al.^11^ have shown a significant relationship between the severity of atherosclerosis on ultrasound and bone density.

Some responsible mechanism has been claimed for association of cardiovascular disease and osteoporosis. Molecular studies have showed that some factors and specific proteins that act in osteogenesis may also affect the formation of calcified atherosclerotic vascular lesions.^4^ Moreover, osteoprotegerin, an effective protein in bone resorption may have a critical role in bone formation. It has been shown that lack of osteoprogerin in rats develops osteoporosis and medial calcification in the aorta.^12^

BAC was first described by Sickles in 1985 as two parallel calcified lines or calcified ring on mammography.^13^ BAC was defined as accumulation of calcium in the medial layer of the peripheral arterioles, called as Mönckeberg medial calcific sclerosis or medial arterial calcification.^14^ Previous studies have shown the relationships between BAC and cardiovascular diseases (CVD), diabetes mellitus (DM), and hypertension (HT).^3^ Reddy et al.^4^ has documented the relationship between reduction in BMD and BAC. In their study, a statistically significant relationship was found between BAC and osteopenia (p<0.01) and osteoporosis (p=0.006).^4^ In our study, a significant relationship was also found between osteoporosis and BAC (p<0.001). However, osteopenia was more common in our BAC (-) group (p<0.001).

There are some limitations of our study. First limitation is small sample size, and secondly, we did not evaluate the effect of hormone replacement therapy (HRT). Despite it has been stated that HRT has anti-resorptive effect on bone and are is accepted as a useful for treatment of osteoporosis, significantly increased levels of BAC were also reported in women who had never used HRT.^15^ The unexplained relationship of BAC, BMD, and HRT may be considered in a novel study.

In conclusion, determination of BAC in routine screening mammography might be helpful in both identifying women with risk of cardiovascular disease and osteoporosis. However, more clinical studies are needed to further clarify the pathophysiological correlation between BAC and osteoporosis.

## References

[1] Topal U, Kaderli A, Topal NB, Ozdemir B, Yesilbursa D, Cordan J (2007). Relationship between the arterial calcification detected in mammography and coronary artery disease. Eur J Radiol.

[2] Kataoka M, Warren R, Luben R, Camus J, Denton E, Sala E (2006). How predictive is breast arterial calcification of cardiovascular disease and risk factors when found at screening mammography?. Am J Roentgenol.

[3] Schnatz PF, Rotter MA, Hadley S, Currier AA, O’Sullivan DM (2007). Hormonal therapy is associated with a lower prevalence of breast arterial calcification on mammography. Maturitas.

[4] Reddy J, Bilezikian JP, Smith SJ, Mosca L (2008). Reduced bone mineral density is associated with breast arterial calcification. J Clin Endocrinol Metabol.

[5] (1994). Assessment of fracture risk and its application to screening for postmenopausal osteoporosis. Report of a WHO Study Group. World Health Organization technical report series.

[6] Yildiz S, Yildiz A, Ertug N, Kaya I, Yilmaz R, Yuksel E (2008). Association of breast arterial calcification and carotid intima-media thickness. Heart and Vessels.

[7] Jorgensen L, Engstad T, Jacobsen BK (2001). Bone mineral density in acute stroke patients: low bone mineral density may predict first stroke in women. Stroke.

[8] Kiel DP, Kauppila LI, Cupples LA, Hannan MT, O’Donnell CJ, Wilson PW (2001). Bone loss and the progression of abdominal aortic calcification over a 25 year period: the Framingham Heart Study. Calcified Tissue Int.

[9] Marcovitz PA, Tran HH, Franklin BA, O’Neill WW, Yerkey M, Boura J (2005). Usefulness of bone mineral density to predict significant coronary artery disease. Am J Cardiol.

[10] Schulz E, Arfai K, Liu X, Sayre J, Gilsanz V (2004). Aortic calcification and the risk of osteoporosis and fractures. J Clin Endocrinol Metabol.

[11] Uyama O, Yoshimoto Y, Yamamoto Y, Kawai A (1997). Bone changes and carotid atherosclerosis in postmenopausal women. Stroke.

[12] Romrell LJ, Bland KI, Bland KI, Copeland EM (1991). Anatomy of the Breast, axilla, chest wall and related metastatic sites. The Breast. Comprehensive Management of Benign and Malignant Diseases.

[13] Sickles EA, Galvin HB (1985). Breast arterial calcification in association with diabetes mellitus: too weak a correlation to have clinical utility. Radiology.

[14] Cetin M, Cetin R, Tamer N (2004). Prevalence of breast arterial calcification in hypertensive patients. Clin Radiol.

[15] Cox J, Simpson W, Walshaw D (2002). An interesting byproduct of screening: assessing the effect of HRT on arterial calcification in the female breast. J Med Screen.

